# Biallelic variants identified in 36 Pakistani families and trios with autism spectrum disorder

**DOI:** 10.1038/s41598-024-57942-x

**Published:** 2024-04-22

**Authors:** Hamid Khan, Ricardo Harripaul, Anna Mikhailov, Sumayah Herzi, Sonya Bowers, Muhammad Ayub, Muhammad Imran Shabbir, John B. Vincent

**Affiliations:** 1 Molecular Neuropsychiatry and Development (MiND) Lab, Campbell Family Mental Health Research Institute, Centre for Addiction and Mental Health, Centre for Addiction and Mental Health, 250 College St, Toronto, ON M5T 1R8 Canada; 2 Department of Biological Sciences, International Islamic University Islamabad, Islamabad, Pakistan; 3 Institute of Medical Science, University of Toronto, Toronto, ON Canada; 4 Department of Psychiatry, UCL, London, UK; 5 Department of Psychiatry, University of Toronto, Toronto, ON Canada

**Keywords:** Consanguinity, Cytogenetics, Neurodevelopmental disorders, Sequencing

## Abstract

With its high rate of consanguineous marriages and diverse ethnic population, little is currently understood about the genetic architecture of autism spectrum disorder (ASD) in Pakistan. Pakistan has a highly ethnically diverse population, yet with a high proportion of endogamous marriages, and is therefore anticipated to be enriched for biallelic disease-relate variants. Here, we attempt to determine the underlying genetic abnormalities causing ASD in thirty-six small simplex or multiplex families from Pakistan. Microarray genotyping followed by homozygosity mapping, copy number variation analysis, and whole exome sequencing were used to identify candidate. Given the high levels of consanguineous marriages among these families, autosomal recessively inherited variants were prioritized, however de novo/dominant and X-linked variants were also identified. The selected variants were validated using Sanger sequencing. Here we report the identification of sixteen rare or novel coding variants in fifteen genes (*ARAP1*, *CDKL5*, *CSMD2*, *EFCAB12*, *EIF3H*, *GML*, *NEDD4*, *PDZD4*, *POLR3G*, *SLC35A2, TMEM214*, *TMEM232*, *TRANK1*, *TTC19*, and *ZNF292*) in affected members in eight of the families, including ten homozygous variants in four families (nine missense, one loss of function). Three heterozygous de novo mutations were also identified (in *ARAP1*, *CSMD2*, and *NEDD4*), and variants in known X-linked neurodevelopmental disorder genes *CDKL5 and SLC35A2*. The current study offers information on the genetic variability associated with ASD in Pakistan, and demonstrates a marked enrichment for biallelic variants over that reported in outbreeding populations. This information will be useful for improving approaches for studying ASD in populations where endogamy is commonly practiced.

## Introduction

Autism spectrum disorders (ASD) are neurodevelopmental and neuropsychiatric conditions with two main symptoms: impaired social communication and repetitive behaviours^1^. Estimated prevalence of ASD is 1/36 among eight-year olds in the US, as of 2020^2^. There is no official data available on prevalence of ASD in Pakistan^3^, however a systematic review of published literature from South Asian countries concluded the prevalence of ASD to be in the range of 0.09–1.07%. There are relatively few studies that have explored the genetic architecture of ASD in Pakistan. Due to its diverse ethnic population, yet high rate of consanguineous marriages, Pakistan is predicted to have a wide range of genetic variants, but with enrichment of disease-related autosomal recessive variants.

ASD is a phenotypically diverse condition, and is associated with a wide range of other disorders and comorbidities, such as intellectual disability (ID), attention deficit hyperactivity disorder (ADHD), epilepsy, Fragile X syndrome, Rett syndrome, anxiety, depression, motor abnormalities, gastrointestinal problems, sleep disorders and in some cases dysmorphic features as well^4^. ASD is also a genetically diverse condition that can result from both inherited and sporadic genetic and genomic variants. There are hundreds of candidate genes reported as risk factors for ASD^5^. 

While studies of ASD have identified copy number variants (CNVs) and point mutations that contribute significantly to the genetic architecture of ASD, the majority of these studies were conducted in outbred populations. This limits the scope of ASD studies, as identification of autosomal recessive (AR) variants would not be favoured, and thus an important part of the genetic architecture of ASD may be under-represented. A few studies that have highlighted AR inheritance as an important component of the genetic architecture of ASD, including a study of consanguineous versus non-consanguineous families in India that suggested consanguinity to be an important risk factor, increasing risk for ASD with an odds ratio of 3.22^6^.

Our recent study of 115 ASD trios from Pakistan, Iran and Saudi Arabia identified 84 candidate variants, the majority (58%) being biallelic (Harripaul et al., MedRXIV)^7^, suggesting a significant enrichment of AR genes in populations where consanguinity is common. Here we focus on 36 ASD families (26 proband/mother/father trios and eight small multiplex families and two proband-parent diad) from Pakistan, using SNP microarray and whole exome sequencing analysis to identify genetic and genomic variants.

## Results

### Family structure

36 families were included in the study, including 26 complete trios, eight multiplex families, and two diads where one parent of a trio was missing. The family structures are presented in tabular form (Table 1). For five of the trios, an unaffected sibling’s DNA was available, but not used, except for Sanger sequencing to check segregation. PLINK relatedness analysis indicated consanguinity for 15 of the families (see Supplementary Table S3). Also, there was possible cryptic relatedness between families PKASD-04 and PKASD-14, however no HBD was shared between the two families.

**Table 1:** Description of families, trios, or diads available for the study. a: DNA from unaffected sister also available; b: affected siblings are monozygotic twins; c: affected twin brother, DNA unavailable, zygosity status of twins unknown; In addition, pedigrees of all multiplex families are shown in Supplementary Materials.

Family type	N	Family IDs
Complete trios, male proband	23	PKASD2, PKASD3, PKASD5, PKASD6, PKASD7, PKASD10, PKASD15, PKASD16, PKASD18, PKASD19^a^, PKASD20, PKASD27, PKASD28, PKASD30, PKASD31, PKASD32, PKASD33, PKASD35, PKASD36^a^, PKASD37, PKASD38^a^, PKASD39, PKASD47
Complete trios, female proband	3	PKASD8, PKASD11, PKASD29
Proband-mother diad (father missing)	2	PKASD1, PKASD21
Multiplex, 2 affected sisters	2	PKASD4, PKASD34^a,b^
Multiplex, 2 affected brothers	4	PKASD14, PKASD22^c^, PKASD25, PKASD26
Multiplex, 1 affected brother, 1 affected sister	1	PKASD13
Multiplex, 2 affected sisters, 1 affected brother	1	PKASD17

### HBD mapping

HBD regions greater than 1 Mb in length for seven multiplex families are listed in Supplementary Table S1. Under the hypothesis of autosomal recessive inheritance, WES data for these seven families focused on homozygous variants within the HBD blocks (HBD could not be run for one of the eight multiplex families, PKASD-22, as microarray data for one of the affected siblings was unavailable). Missense variants were identified in the following genes, and subsequently validated, and segregation confirmed: *EIF3H*, *TRANK1*, *ZNF292* (see Table 2; Supplementary Table S3). In silico predictions of effect of the amino acid substitution are listed in Table 3.

**Table 2:** Candidate variants: Genomic coordinates are given relative to GRCh37/hg19 build. gnomAD minor allele frequency (MAF) v2.1.1 is provided, with number of homozygotes or hemizygotes for X-linked (XL) genes given in parentheses. * indicates the variant is located within an HBD block (in a multiplex family). ClinVar reports were identified from other studies or diagnostic clinics for *TTC19* (recorded as pathogenic and likely pathogenic), GML (benign), and *POLR3G* (variant of uncertain significance) (accessed 19 Jan 2024).

Family ID	Sex	Chromosome	Gene	mRNA variant	Protein variant	Mutation type	Mutation effect	gnomAD MAF all	gnomAD MAF S. Asian
PKASD-14	M	3:36905962T > C*	*TRANK1*	NM_001329998.2:c.1088A > G	p.Asn363Ser	Homo	Missense	2.99E−05 (0)	2.00E−04 (0)
		3:36931409T > C*	*TRANK1*	NM_001329998.2:c.818A > G	p.Glu273Gly	Homo	Missense	7.62E−04 (0)	4.84E−04 (0)
PKASD-18	M	X:18646620A > G	*CDKL5*	NM_003159.3:c.2626A > G	p.Ile876Val	XL	Missense	0	0
PKASD-19	M	X:48762117C > G	*SLC35A2*	NM_001042498.3:c.1069G > C	p.Gly357Arg	XL	Missense	1.1E−05 (1)	5.304E−05 (1)
PKASD-26	M	17:15909789C > T	*TTC19*^a^	NM_017775.4:c.583C > T	p.Gln195*	Homo	Stop gain	3.18E−05 (0)	3.266E−05 (0)
		6:87971373G > A*	*ZNF292*	NM_015021.3:c.8026G > A	p.Asp2676Asn	Homo	Missense	3.891E−04 (0)	3.486E−03 (0)
		8:117767987G > C*	*EIF3H*	NM_003756.3:c.50C > G	p.Ser17Cys	Homo	Missense	7.562E−05 (0)	6.206E−04 (0)
		8:143921881A > G*	*GML*^b^	NM_002066.3:c.28A > G	p.Met10Val	Homo	Missense	4.95E−05 (0)	1.96E−04 (0)
PKASD-32	M	15:56207541C > T	*NEDD4*	NM_001284338.2:c.1489G > A	p.Asp497Asn	De novo	Missense	0	0
PKASD-37	M	3:129147293C > G	*EFCAB12*	NM_207307.3:c.39G > C	p.Leu13Phe	Homo	Missense	1.871E−4 (0)	1.489E−3 (0)
		X:153070213G > A	*PDZD4*	NM_001303512.2:c.923C > T	p.Pro308Leu	XL	Missense	5.5E−06 (0)	0
		11:72410069C > T	*ARAP1*	NM_001040118.3:c.2522G > A	p.Trp841*	De novo	Stop gain	0	0
PKASD-39	M	1:34082538C > T	*CSMD2*	NM_001281956.2:c.5984G > A	p.Arg1995Gln	De novo	Missense	0	0
PKASD-47	M	2:27259973G > A	*TMEM214*	NM_017727.5:c.937G > A	p.Gly313Ser	Homo	Missense	1.21E−04 (0)	8.496E−04 (0)
		5:89781487C > A	*POLR3G*^c^	NM_006467.3:c.103C > A	p.Pro35Thr	Homo	Missense	1.429E−04 (0)	2.973E−04 (0)
		5:109973924T > C	*TMEM232*	NM_001039763.4:c.476A > G	p.Tyr159Cys	Homo	Missense	0	0

**Table 3:**
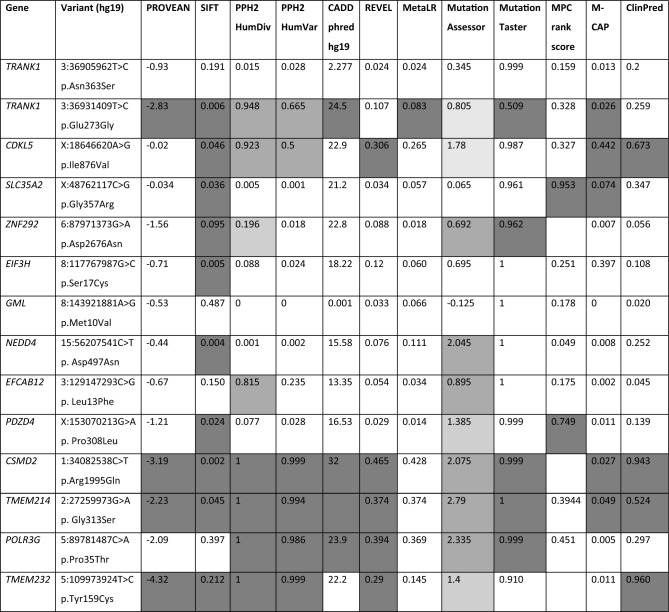
In silico predictions missense variants. Prediction scores (to 3 decimal places), generated using dbNFSP (http://database.liulab.science/dbNSFP), predictions within the benign or neutral range are unshaded, light grey for low probability of damaging, medium grey for probably damaging, and dark grey shading indicates within the damaging range.

### Whole exome sequencing

#### PKASD-14

*Clinical findings* This family was enrolled from Lahore, Punjab province. The family has had consanguineous marriages for at least the last two generations, including the parents of the affected siblings, but with no previous family history of ASD. The two sons both show clinical features of ASD. The boys, IV: 1 and IV:2, were aged six and seven years at enrollment, respectively. Both of the probands display diverse clinical manifistations. Individual IV:1 showed moderate autistic features (non-verbal, no reciprocal communication/social interaction; stereotypic behaviours), no ID, obsessive compulsive disorder, anxious/fearful, occasionally aggressive, insomnia, no hyperactivity, no seizures, occasional gait abnormality. Biochemical analysis showed possible vitamin D deficiency, low levels of pancreatic elastase and secretory IgA, high lysozyme levels; organic acid tests showed high tartaric acid and low serotonin; urine levels suggested high levels of toxic metals (lead, mercury, and cadmium). Individual IV:2 was diagnosed with mild to moderated ASD (non-verbal, no reciprocal communication/social interaction; stereotypic behaviours), possible mild cognitive deficit, difficulty concentrating, occasionally aggressive, difficulty getting to sleep, no hyperactivity, no seizures, low appetitite, along with decreased muscle tone. Biochemical analysis showed high lead, arsenic and barium levels, and possible vitamin C deficiency. Blood samples were collected from both affected individuals and parents (III:1 III:2) for genetic studies.

**Figure 1:**
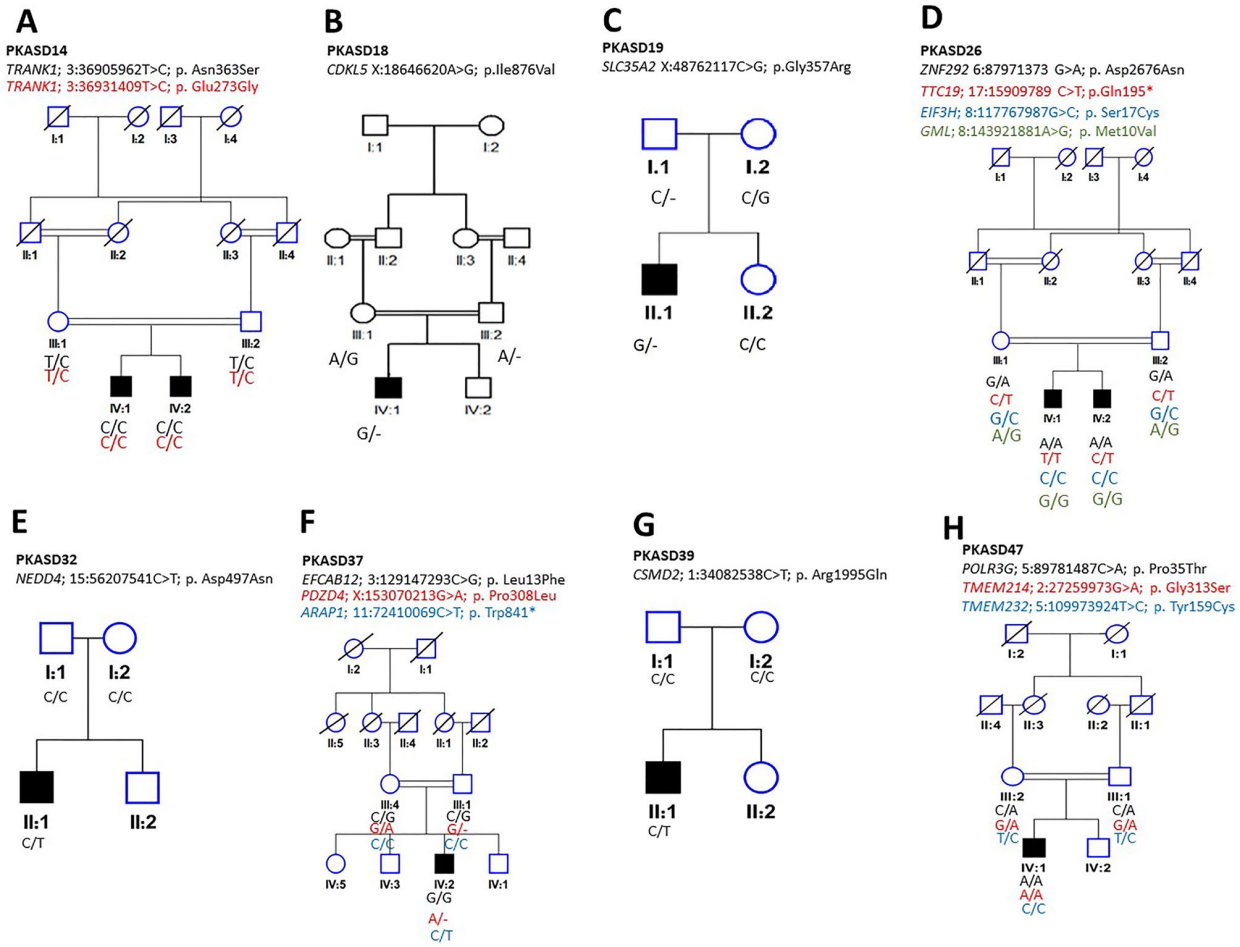
Pedigrees and variant segregation for the sixteen variants in eight families reported. Genomic coordinates provided are using GRCh37/hg19.

*Genetic findings* Through microarray analysis and HBD mapping, we identified nine blocks of shared HBD over 1 Mb in length, accounting for over 99 Mb of the autosome. Within a 19.8 Mb block on chromosome 3, we identified a two homozygous missense variants in the gene *TRANK1*: NM_001329998.2:c.1088A > G; p.Asn363Ser, and NM_001329998.2:c.818A > G; p.Glu273Gly*.*Segregation is shown in Fig. 1A. In silico predictions suggest the former to likely be benign (12 of 12 algorithms), but the latter to be damaging (9 of 12 algorithms; see Table 3).

#### PKASD-18

*Clinical findings* PKASD-18 is a family of Punjabi origin from Islamabad, Pakistan with consanguineous marriages in the last two generations, and with no known family history of ASD. The family has two sons, the affected child is a 7-year-old boy and the other is 4 years old and neurotypical. The affected child was diagnosed with ASD at the age of 42 months by a paediatrician in Pakistan. The affected individual shows a diverse range of clinical manifestations including, poor eye contact, nonverbal communication (just shouting), social isolation, intellectual disability, severe aggression and self-injuring behaviour, with severity increasing with age. No epilepsy was present.

*Genetic findings* In PKASD-18 We identified a novel X-linked hemizygous missense variant at NM_003159.3:c.2626A > G, NP_003150.1:p.Ile876Val in cyclin-dependent kinase-like 5 (*CDKL5*) gene. The mutation was absent in gnomAD, including in the South Asian population. IGV and Sanger sequencing support maternal inheritance of the variant. Segregation is shown in Fig. 1B. *CDKL5* is a known gene for developmental and epileptic encephalopathy 2 (MIM 300672), also known as CDKL5 deficiency disorder (CDD), which has some clinical overlap with Rett syndrome (MIM 312750). Seven of the 12 prediction algorithms indicated the variant to be damaging (Table 3).

#### PKASD-19

*Clinical findings* The affected individual shows a diverse clinical manifestations including, poor eye contact, poor communication, social isolation, intellectual disability, seizures, severe aggression and self-injuring behaviour. This family was enrolled from Lahore District, Punjab, Pakistan. The parents have a non consanguineous marriage with a history of epilepsy and developmental delay from both sides. The family has a total of two children (one son and one daughter). The affected individual is an older 13-year-old boy, while the sibling is neurotypical. The affected boy was diagnosed with ASD at the age of 4 years by a certified paediatrician in Pakistan. Epilepsy was also reported for this individual.

*Genetic findings* An X-linked missense variant at NM_001042498.3:c.1069G > C, NP_001035963.1:p.Gly357Arg in the solute carrier family 35 member A2 (*SLC35A2*) gene was identified In PKASD-19. Segregation of the variant in this pedigree was verified by Sanger sequencing, with the father hemizygous wild type and mother heterozygous, while the affected individual is hemizygous for the variant. Segregation is shown in Fig. 1C. Two alleles were noted in gnomAD (including one hemizygote in the South Asian population), indicating that it is a very rare variant. In silico predictions for this missense variant are summarized in Table 3. Mutations in *SLC35A2* have previously reported for a developmental and epileptic encephalopathy 22 (DEE22), or congenital disorder of glycosylation type IIm (CDG IIm; MIM 300896).

#### PKASD-26

*Clinical finding* PKASD-26 have had consanguineous marriages in last two generations, including the parents of the affected individuals, and with no family history of ASD. The family has two sons, both of whom were diagnosed with ASD. Child 1 (IV: 1), aged 14-year, is reported as having moderate ASD features along with movement imbalance and difficulty with chewing (dysphasia), as well as intellectual ability declining with age. The second child (IV: 2), a 12-year-old brother, was diagnosed with mild to moderate ASD, and with no comorbid phenotypes.

*Genetic findings* HBD mapping indicated 14 blocks of autozygosity > 1 Mb in length (Table S1) in PKASD-26. The following WES variants identified within the HBD blocks are listed in Table 3. Within a ~ 24 Mb HBD block, a homozygous missense variant was identified in the gene *ZNF292*, at NM_015021.3:c.8026G > A, p.Asp2676Asn, in both affected individuals (IV:1 and IV:2) of PKASD-26. Also, within a 14 Mb block on chromosome 8, we identified a homozygous missense variant in *EIF3H*: NM_003756.3:c.50C > G; p.Ser17Cys. Within the same HBD block, we identified a homozygous missense variant in *GML*: NM_002066.3:c.28A > G; p.Met10Val. The variants in *ZNF292*, *GML*, and *EIF3H* were homozygous in both of the affected individuals and heterozygous in the parents. We also identified a rare homozygous stop-gain variant in *TTC19* in child 1 (IV:1), outside of HBD blocks, at NM_017775.4:c.583C > T; p.Gln195*. Sanger validation showed the variant to be heterozygous in child 2 (IV:2) and in both of the parents. All four variants have very low frequencies in gnomAD (*ZNF292*: 3.891E-04, *EIF3H*: 7.652E-05, *GML*: 4.95E-05, and *TTC19*: 3.184E-05). Segregation is shown in Fig. 1D. Multiple in silico predictions for these missense variants are summarized in Table 3.

#### PKASD-32

*Clinical findings* PKASD-32, a non-consanguineous trio with no prior family history of ASD, is from Karachi, Sind Province, Pakistan. The family has two sons: the affected child is an 8.5-year-old boy and the other is 5 years old and neurotypical. Common behavioral and phenotypical abnormalities noted were social and communication delay, social isolation, repetitive behavior, poor eye contact, sensory issues, poor social reciprocal interest. The child showed significant impairments with his social interactions/interactions and significant developmental delay in most areas, and meeting criteria for ASD on DSM V. He was diagnosed with ASD by a certified developmental and behavioral pediatrician from the history provided, the developmental profile, the Childhood Autism Rating Scale (CARS), and functional behavior analysis.

*Genetic findings* In PKASD-32 a de novo missense mutation was identified through WES at NM_001284338.2:c.1489G > A, p.Asp497Asn in Neural precursor cell Expressed, Developmentally Down-regulated 4 (*NEDD4*) gene. Segregation of the variant in this pedigree was confirmed by Sanger sequencing (Fig. 1E). The variant was not present in gnomAD. In silico predictions for this missense variant are summarized in Table 3.

#### PKASD-37

*Clinical findings* This family was enrolled from Charsadda district, Khyber Pakhtunkhwa (KPK) Pakistan. The parents had a consanguineous marriage, and there is no history of ASD in the family, however visual impairment was reported to be common in the family. The family has three sons and one daughter. The affected child is the older 11-year-old boy with mild to moderate ASD and with progressive visual impairment. The other siblings were reported as neurotypical. DNA from the siblings was unavailable for study.

*Genetic findings* In PKASD- 37 three missense variants were identified by WES. The first is a homozygous missense at NM_207307.3:c.39G > C, p.Leu13Phe in EF-hand calcium-binding domain-containing protein 12 (*EFCAB12*) gene. The change was confirmed by Sanger sequencing, and segregated in family. The second identified variant is an X-linked, maternally inherited missense variant at X: 153070213G > A, NM_001303512.2:c.923C > T, p.Pro308Leu in PDZ domain-containing 4 (*PDZD4*) gene. The variant was confirmed by Sanger sequencing and segregated in the family (Fig. 1F). The third variant identified is a de novo missense mutation at NM_001040118.3:c.2522G > A, p.Trp841* in ArfGAP with RhoGAP domain, ankyrin repeat, and pleckstrin homology domain 1 (*ARAP1*) gene (also known as *CENTD2*). The mutation was heterozygous in the proband and wild type in both of the parents. The variants were either very rare or not present in gnomAD. Multiple approaches for predicting the outcome of these missense variants are summarized in Table 3.

#### PKASD-39

*Clinical findings* PKASD-39, a non-consanguineous trio with no prior family history of ASD, was enrolled in Islamabad, Pakistan. The family has two children, an older 8 year-old autistic boy and a 1 year old girl reported as developing normally. The affected child was diagnosed with ASD at the age of 4 years by a paediatrician in Pakistan.

*Genetic findings* In PKASD-39 we identified a novel de novo missense mutation at NM_001281956.2:c.5984G > A, p.Arg1995Gln in CUB and Sushi multiple domains 2 (*CSMD2*) gene. Segregation was validated by Sanger sequencing (Fig. 1G). The variant was absent in gnomAD.

#### PKASD-47

*Clinical findings* Family PKASD-47 have two children: the elder one (IV: 1) is an eight year old son who is diagnosed with ASD, and the younger one (IV: 2) is a five year old son reported as neurotypical. The developmental and communication milestones of the affected individual were delayed. The major clinical manifestations included ASD, developmental delay, nystagmus, and seizures. Electroencephalogram (EEG) indicated focal seizure disorder arising from the occipital regions (bilateral). Biochemical tests did not show any abnormality. MRI was also performed, and the major finding was reported as metachromatic leukodystrophy (MLD; MIM 250100), however, although behavioural and cognitive difficulties were present, ataxia, upper motor signs and neuropathy, all features of juvenile onset MLD, were not identified.

*Genetic* *findings* In PKASD- 47 three homozygous missense variants were identified by WES, including in *POLR3G* (NM_006467.3:c.103C > A, p.Pro35Thr), in *TMEM232* (NM_001039763.4:c.476A > G, p.Tyr159Cys), and in *TMEM214* (NM_017727.5:c.937G > A, p.Gly313Ser). Sanger sequencing confirmed segregation for all three variants (Fig. 1H). These variants are either very rare or not present in gnomAD, and with zero homozygotes (see Table 2). The in silico predictions for these variants were mainly damaging (see Table 3). No variants were identified in known MLD genes (e.g. *ARSA*, *PSAP*).

### CNV analysis

No homozygous loss CNVs were identified that were corroborated by WES read evidence using IGV. Also, no loss/gain CNVs were identified with a CNVPartition score over 100 that did not overlap < 50% with a known CNV in the DGV database.

### Study limitations

It is important to acknowledge that the present study and methodology employed would be unable to detect certain types of variants that may be important to ASD. These include deep intronic variants which may activate a cryptic splice site or impact the regulation of gene expression, similarly intergenic variants, also some variants within highly GC coding regions, due to the nature and biases of the WES procedures. The WES variant analysis is also insufficiently sensitive to the presence of simple tandem repeat expansions, such as in the FMR1 gene that are responsible for fragile X syndrome (MIM 300624), which have been found in many individuals with ASD. Also, the microarray CNV analysis would be unable to detect inversions or balanced translocations.

## Discussion

There are relatively few studies that have explored the genetic architecture of ASD in Pakistan. Due to its diverse ethnic population, yet high rate of endogamy, Pakistan is predicted to have a wide range of genetic variants, but with enrichment of disease-related autosomal recessive variants. In this study of 36 small ASD families, we identified ten candidate biallelic variants, three de novo autosomal (dominant) mutations, and three maternally inherited X-linked variants. For the other families, our analysis either identified no variants that satisfied our filtering criteria, or the identified variants did not validate.

We were able to identify multiple blocks of HBD in seven of the eight multiplex families (Supplementary Table S1). Putative biallelic candidate missense variants were identified within these regions for families PKASD14 (*TRANK1*) and PKASD26 (*ZNF292* and *EIF3H*). *TRANK1*, encoding tetratricopeptide repeat-and ankyrin repeat-containing protein 1, is one of the most replicated genome-wide association study (GWAS) findings for bipolar disorder^8^ (e.g. Mullins et al., 2021; p = 1.5E-15). One of the other most consistent GWAS findings for bipolar disorder is another ankyrin gene, *ANK3*^8^ (e.g. Mullins et al., 2021; p = 1.6E-11). Biallelic variants in *ANK3* are responsible for an autosomal recessive form of ID, MRT37 (MIM 615493). Thus, this could be another bipolar disorder-associated ankyrin gene linked autosomal recessive neurodevelopmental disorders. It should also be noted that excessive urine lead levels were reported in both PKASD14 affected siblings, which also could be a potential risk factor for neurodevelopmental disorder. The elevated tartaric acid and decreased serotonin levels reported in PKASD14 IV:1 are unlikely to be related to ASD symptoms; tartaric acid levels are more related to diet, and serotonin levels related to depression (albeit controversially)^9^.

In trio PKASD-18, which has one autistic and one neurotypical son, we identified a novel X-linked maternally inherited missense mutation, NM_003159.3:c.2626A > G; p.Ile876Val in *CDKL5* (Cyclin-Dependent Kinase-Like 5), a known gene for an infantile epileptic encephalopathy and Rett syndrome-like disorder (MIM 300672), which, like Rett syndrome, affects girls almost exclusively. However missense variants in *CDKL5* have also been reported previously for ASD. For instance, Codina-Solà, et al.^10^ found a maternally inherited missense variant (p.Pro647Leu) in *CDKL5* in a male child affected with ASD. There are also a number of candidate missense hemizygous variants present in ASD males in the MSSNG database, as well as a frameshifting deletion in a heterozygous female (Supplementary Table S3). Variants that cause CDKL5-deficiency disorder (CDD; MIM 300672) in females would be expected to have much more severe clinical consequences in males. The PKASD-18 proband, in addition to ASD, presented with ID, along with aggression and self-injurious behaviour. He was also non-verbal, which is commonly noted in CDD individuals. However, there were no signs of epilepsy, which is present in 90% of CDD girls in the first three months of life, nor the gross motor impairment present in almost all CDD girls^11^. Affected CDD males are typically unable to walk, with or without assistance. Thus, to cause a milder phenotype in males, missense changes would be expected to be relatively mild and hypomorphic. For the PKASD-18 mutation, Ile876Val is predicted to be deleterious by Polyphen2, M-CAP, ClinPred, REVEL, and SIFT, whereas other prediction algorithms rank the change as neutral, low or tolerated (Table 3). It is also possible that, while CDD mutations result in loss-of-function of CDKL5, the Ile876Val mutation results in a functional gain.

In PKASD-19 we identified a maternally inherited X-linked missense variant in *SLC35A2*. *SLC35A2* encodes the UDP-galactose transporter protein, which is in responsible for carrying UDP-galactose, which plays an active role in protein glycosylation. These mutations may impair the UDP-galactose transporter's ability to operate normally, which could have an effect on glycosylation procedures and perhaps have an effect on brain function and ASD development. Mutations in *SLC35A2* are listed as the cause of congenital disorder of glycosylation type IIm (CDG2M) or developmental and epileptic encephalopathy-22 (MIM 300896). Mutations are typically found in female patients and de novo, however there are a few male cases reported, e.g. de novo hemizygous mutation p.Leu154Pro in a boy with CDG2M, another boy with CDG with the de novo hemizygous mutation p.Lys78Arg^12^, and a study reported a maternally inherited rare variant (p.Val258Met) in *SLC35A2* in a male with Rett-like phenotype^13^. Epilepsy is a major comorbid phenotype in the PKASD-19 proband. Comparing the predicted effects of missense mutations in males diagnosed with neurological impairments with or without skeletal abnormalities listed in Ng et al., 2019^14^ and Rett-like symptoms^13^, the current variant in PKASD-19 has fewer algorithms designating it as deleterious (Table 3). In addition, a single hemizygote for p.Gly357Arg was present in the gnomAD control exome dataset, in the South Asian cohort. Thus, functional characterization using a biochemical assay developed to assess SLC35A2-dependent UDP-galactose transport activity in patient-derived fibroblasts^14^ may be necessary to determine whether this mutation is related to the phenotype in the proband. Interestingly, in the MSSNG ASD database there are two affected brothers (ID# 5–5015-003 and 005) hemizygous for a missense variant NM_005660:c.109T > C: p.Y37H in *SLC35A2*, that is maternally inherited, but not present in gnomAD controls.

PKASD-26 is a consanguineous family with two ASD siblings (IV: 1 & IV: 2). HBD analysis gives 14 blocks of shared homozygosity greater than 1 Mb in length, including a ~ 24 Mb block on chromosome 6 (6q21). Within this block we identified a homozygous missense variant, p.Asp2676Asn, in *ZNF292*, validated in both (IV: 1 & IV: 2) autistic individuals and heterozygous in both parents. Heterozygous variants (typically de novo) in *ZNF292* have been reported as a recurring cause of autosomal dominant intellectual developmental disorder 64 (MRD64; MIM 619188^15^. The families included in the 2020 study included a Pakistani ASD trio (also see^7^). *ZNF292*, encoding a zinc finger protein, is listed as a SFARI autism gene designated 1S (syndromic). We observed movement imbalance, dysphasia, and declining intellectual abilities in child1 (IV: 1) of PKASD-26. We also identified a rare homozygous loss-of-function (LoF) variant in *TTC19* in child one (IV: 1) but not child 2 (IV:2), NM_017775.4:c.583C > T, p.Gln195*, which was not in a block of HBD. Variants in *TTC19* have been associated with mitochondrial complex III deficiency, nuclear type 2 (MIM 615157)^16^. Symptoms include motor disability, ataxia, apraxia, dysarthria, muscle weakness, exercise intolerance, and respiratory failure^17^. Patients typically also develop cognitive impairment, and onset of symptoms can range from childhood to adulthood^18^. This would suggest that many of the comorbid features present in sibling IV:1 that are absent from IV:2 are likely due to *TTC19*, whereas the milder ASD-related symptoms shared by both may be due to *ZNF292*. Most of the algorithms used predict the effect of the missense substitution to be tolerated, however, given that this is the only report to date of a homozygous missense variant, and all previously reported disease-related rare variants are heterozygous (ClinVar, accessed 26 Jan 2024), and since both parents are unaffected, it is still quite possible that the variant can only exert an effect in the homozygous form. Importantly, cross-referencing with the MSSNG database of ASD trios, while no de novo variants could be confirmed, another homozygous missense variant, NM_015021:c.6946C > T:p.Arg2316Trp, was present in an affected male (ID# AU2688301), with a control population minor allele frequency of 1.759E-04 (gnomAD v2.1.1, accessed Aug 2023; no homozygotes). Homozygous missense variants were also identified in *EIF3H*, and *GML*, in HBD regions on chromosome 8 (Table 2), but are predicted by 11/12 and 12/12 algorithms, respectively, to be tolerated/benign (Table 3).

A de novo missense mutation was identified in PKASD-32 in *NEDD4*. NEDD4 (Neural precursor cell Expressed, Developmentally Down-regulated 4) is a gene that encodes for an E3 ubiquitin ligase, which plays a role in protein degradation and cellular processes. *NEDD4* has been implicated in various biological pathways and cellular processes that are relevant to neurodevelopment and synaptic function^19^. It is involved in regulating the levels of specific proteins within cells, including ion channels and receptors, which are critical for proper neuronal communication and signaling. Disruptions in these processes could potentially contribute to the development of ASD, however, predictions for the amino acid substitution would suggest the variant is likely benign (Table 3).

In PKASD-37 three missense variants in three different genes including a homozygous variant in *EFCAB12*, an X-linked variant in *PDZD4* and a de novo mutation in *ARAP1*. *EFCAB12* is a calcium-binding protein that is strongly expressed in brain, and found in soma and synapse in neurons (https://www.proteinatlas.org/ENSG00000172771-EFCAB12/brain; accessed 26 Jan 2024). A study found a loss-of-function variant in *EFCAB12* in a patient with unilateral kidney anomaly, and a number of extrarenal symptoms, such as neurodevelopmental delay, epilepsy, and corpus callosum^20^. *ARAP1* (also known as *CENTD2*) participates in a number of physiological functions, including membrane trafficking, cytoskeletal organization, and signal transduction. It encodes a protein that controls the activity of small ATPase’s, including the Arf and Rho proteins, which are crucial for cellular processes and synaptic formation^21,22^. This protein serves as an ATPase-activating protein (GAP). The ARAP1 protein (also known as CENTD2) is involved in regulating cellular processes such as cell adhesion, migration, and signaling^23,24^. *PDZD4*, encoding PDZ domain-containing protein 4, is located on the X chromosome. The involvement of *PDZD4* in ASD is not yet well-established but a cohort study of 285 ASD and schizophrenia patients revealed a maternally inherited hemizygous missense variant, p.Asp326Asn, in *PDZD4* with disruption of protein function predicted^25^. In the MSSNG dataset two males have missense variants in *PDZD4* for which no alleles are present in gnomAD control data (Supplementary Table S3).

In PKASD-39 we identified a novel de novo missense mutation in *CSMD2*. The CSMD2 protein includes CUB and Sushi domains, which function in protein–protein interactions, and CSMD2 is involved in a number of biological functions, such as immune system responses response, synaptic function, and cell adhesion. Interestingly, there have been reports of genetic association between *CSMD2* and schizophrenia^26^ and its paralogue *CSMD1* with cognitive function^27^. The ASC dataset includes a single individual with a de novo frameshifting deletion in *CSMD2* (c.24delC; p.Gly9Alafs*303).

In trio PKASD47 we identified three homozygous missense variants in *POLR3G*, *TMEM232*, and *TMEM214*. *POLR3G* encodes for the RNA polymerase III subunit G protein, which is a component of the RNA polymerase III enzyme. RNA polymerase III is responsible for transcribing genes that encode for various types of small RNA molecules, including transfer RNA (tRNA), 5S ribosomal RNA (rRNA), and other small non-coding RNAs^28^. Although the significance of *POLR3G* variants in ASD has not been well investigated, several studies have linked neurodevelopmental disorders to variants in genes associated to the transcriptional machinery, including parts of RNA polymerase III^29–32^. *TMEM214* and *TMEM232* are both genes that code for transmembrane proteins, neither of which have been associated previously with ASD or other clinical disorder, although deficiency of *Tmem232* in male mice causes abnormalities in sperm flagellum, leading to infertility^33^. All three variants have six or more (out of 12) algorithms predictive of damaging/pathogenicity. However, of the three, *POLR3G* shows highest transcription in brain versus other organs, and *TMEM232* the lowest, with expression mainly in the fallopian tube (females) and testes (males) (proteinatlas.org). Homozygous knockout of *Polr3g* in mice leads to preweaning lethality (https://www.mousephenotype.org). Thus, we postulate that *POLR3G* is the most likely candidate variant, but with *TMEM214* also a possible contributory factor. The ASC dataset includes 3 individuals with de novo missense variants in *TMEM214*.

In conclusion, through the study of 36 small families, we propose candidate biallelic variants in the genes *TRANK1* (Glu273Gly), *ZNF292*, *EFCAB12*, *POLR3G*, and *TMEM214*, variants in the X-linked genes *SLC35A2*, *PDZD4*, and *CDKL5*, and de novo autosomal mutations in *NEDD4*, *ARAP1*, and *CSMD2*, as putative ASD-related variants. Corroboration in ASC and MSSNG datasets supports *CDKL5*, *SLC35A2*, *ZNF292*, *PDZD4*, *CSMD2*, and *TMEM214* (Supplementary Table S3). Although there are no specific studies of ASD in consanguineous families from Pakistan, and given the frequent comorbidity between ASD and ID, we cross-referenced with biallelic variants reported in ID families from Pakistan^34–37^, but no overlap was identified. Further support from additional datasets as well as from functional studies will be required to fully confirm these findings. It is also worth noting that, for nine out of the ten biallelic variants reported in this study (Table 2), there was clear consanguinity, with parents likely to be between second and third degree relatives (Supplementary Materials Table S4), whereas for the six other variants identified (X-linked or de novo), five were in apparently non-consanguineous families and one where there was possible distant relatedness between the parents (PKASD-18). This supports the assertion that, for ID families from populations with high frequency of consanguineous marriages, biallelic variants are the major genetic cause^38^, and that this may also be true for ASD.

## Methods

### Ethical approval and legislative obligations

This study was approved by the Research Ethics Committee, Department of Biological sciences, International Islamic University, Islamabad and also from the research ethics board of the Centre for Addiction & Mental Health (#2008/118), Toronto, and in accordance with the Declaration of Helsinki. Prior to this study, written informed consents were signed by all study participants or their parents/guardians.

### Ascertainment and enrolment of ASD families

Families affected with ASD, including affected singletons plus parents as well as families with multiple affected individuals, were enrolled from different areas of Pakistan. A detailed physical examination was performed before the collection of blood samples of all affected individuals. Detailed family history and related clinical features and physical abnormalities were observed and noticed. All affected individuals were diagnosed by a certified medical specialist, using the Autism Diagnostic Observation Schedule-2 (ADOS-2)^39^ tool, except for family PKASD-39 for which the Childhood Autism Rating Scale™ 2 (CARS™2)^40,41^ was used, following DSM V^42^ diagnostic guidelines. Family pedigrees were drawn by using Cyrillic software (https://www.apbenson.com/software). Family members with no neurodevelopmental or psychiatric diagnosis were not assessed, and are considered for our purposes as neurotypical. DNA from study participants was extracted using standard salting-out methods^43^ or by using Qiagen DNA extraction kit (Qiagen; Hilden, Germany) from whole blood. A standard amelogenin assay (Thermo Fisher Scientific, Waltham, MA) was run, as *per* manufacturer’s recommendations, to confirm the assigned sex of the DNA sample.

### Microarray and exome sequencing

Homozygosity mapping and CNV analysis was performed using data from the Illumina Human Infinium CoreExome-24 v1.4 BeadChip microarrays. These arrays include over 567,000 fixed markers, including 291,536 SNPs. CNV analysis was performed using the Illumina GenomeStudio 2.0 software with CNVpartition plugin. The confidence scores are generated using a likelihood-based algorithm. Default cut-off thresholds (consecutive probes ≥ 3; CNV score ≥ 35; minimum stretch of homozygosity: 1,000,000 bp), however, for analysis purposes we used a more stringent CNV confidence score cut-off > 100. CNVs were compared against the Database of Genomic Variants (DGV) to screen for variants that showed 30% or lower overlap with DGV control variants, or were not in the database^44^. A detailed comparison of this and other CNV algorithms is given by Dellinger et al^45^. Evidence of homozygous loss CNVs was cross-references with WES data, using the Integrated Genome Viewer (IGV: https://software.broadinstitute.org/software/igv/)^46^. CNVs with CNVPartition scores > 100, genic and exonic, were cross-checked for lack of overlap with CNVs in control populations using the Database for Genomic Variants (http://dgv.tcag.ca/dgv/app/home), including gnomAD and 1000 Genomes Phase 3 controls. Regions of homozygosity-by-descent were mapped for multiplex families using homozygosityMapper (www.homozygositymapper.org)^47^. PLINK analysis was performed (PLINK 1.9) on microarray genotypes to check inter- and intra-family relatedness, through identity-by-descent (IBD) analysis using PI_HAT scores, and to compare degree of consanguinity by looking at the proportion of the genome in runs-of-homozygosity (RoH), as described by McQuillan et al., 2008^48^.

WES was performed using the Thruplex DNA-Seq (Rubicon Genomics) Library Preparation Kit with the Agilent SureSelect V5 Exome Capture kit. DNA was sheared using the Covaris ME220 Focused-Sonicator to ensure that the sheared DNA size range was ~ 200 bp. DNA samples were then analyzed using the Agilent 2100 Bioanalyzer System for fragment length distribution and quantification. Library preparation and adapter ligation was performed using the Thruplex DNA-seq kit. All trios and available family members were sequenced on the Illumina NovaSeq sequencing system.

Sentieon® Genomics software was used to convert FastQ files to VCF and BAM files. The VCF files generated for each family were imported into VarSeq® software for annotation. Varseq was also used to compute exome coverage using quality-filtered pileup depth, using targeted region coverage. Sample level statistics were generated such as transition to transversion ratio (TiTv), variant counts, and kinship coefficient. Sequencing data was analyzed using Golden Helix VarSeq™ software with appropriate filters to identify rare, de novo variants, homozygous autosomal variants, X-linked variants, or those inherited in a compound heterozygous manner. All variants identified were checked, firstly to check if they passed the quality metrics as set through VarSeq, to filter out poor variant calls, and minor allele frequency (MAF) in gnomAD (https://gnomad.broadinstitute.org/) of ≤ 1 × 10^–3^ for autosomal recessive, or ≤ 1 × 10^–5^ for autosomal dominant/de novo, prioritizing variants with zero homozygotes in the gnomAD non-Neuro Cohort. For biallelic variants, as the MAF used was on the conservative side, we reanalyzed the data using less stringent values (1 × 10^–3^, 1 × 10^–2^), to see if any variants in known ASD/ID genes were missed under the more stringent analysis. WES reads were then viewed through IGV genome viewer, and then validated by Sanger sequencing in all available family members to confirm its segregation with the disease. Primer sequences for Sanger validation are provided in Supplementary Materials (Table S2). Sequence reads were aligned to the human genome reference sequence [hg19] to observe base pair changes using FinchTV software (Geospiza Inc., Seattle, WA).

Candidate variants were first compared to SNP databases (dbSNP: https://www.ncbi.nlm.nih.gov/snp), in ClinVar (https://www.ncbi.nlm.nih.gov/clinvar), ClinGen https://clinicalgenome.org), and allele frequency using gnomAD (https://gnomad.broadinstitute.org/).

### Homozygosity-by-descent (HBD) mapping

Microarray genotypes from members of the seven families that had two or more affected individuals were extracted using Illumina GenomeSuite software in PLINK format, and uploaded to www.homozygositymapper.org. Chromosomes were inspected at the genotype level for HBD blocks, and for haploidentity within HBD blocks.

### Prediction of effect for missense variants

In order to predict whether candidate missense variants are likely to be damaging to the protein function, and thus potentially disease related, in addition to prediction tools available through Golden Helix VarSeq™ we used a battery of prediction algorithms, including SIFT, PolyPhen2, CADD-Phred, PROVEAN, REVEL, MutationAssessor, and MPC, using the dbNFSP tool (http://database.liulab.science/dbNSFP), version 4.4a^49,50^.

### Cross-referencing with ASD whole exome/genome sequencing datasets

The candidate genes emerging from our study were cross-referenced with the MSSNG dataset ((N > 11,500 individuals; research.mss.ng; accessed Aug 2023), as well as the Autism Sequencing Consortium (ASC) dataset (asc.broadinstitute.org; accessed Aug 2023). High quality coding change variants, either de novo, homozygous (autosomal), or hemizygous (X-chromosomal) that were rare/absent in the gnomAD control datasets (accessed Aug 2023), and with no hemizygotes or homozygotes among the gnomAD controls, were noted (Supplementary Table S3).

### Supplementary Information

Supplementary Information.

## Data Availability

Information on the variants reported here has been submitted to the ClinVar database housed by NCBI: ClinVar submission # SUB13804565. Other data related to the variant analysis is included in the supplementary files. Raw and processed sequence and microarray data will be made available upon reasonable request.
