# Supplementary material for: Biallelic variants identified in 36 Pakistani families and trios with autism spectrum disorder
Source: Sci Rep. 2024 Apr 22;14:9230. doi: 10.1038/s41598-024-57942-x (PMC11035605; doi:10.1038/s41598-024-57942-x)
Supplement: Supplementary file 1 — Supplementary Information. [file 41598_2024_57942_MOESM1_ESM.docx]

**Supplementary Materials**

**Table S1: Homozygosity-by-descent (HBD) regions for seven (out of eight) multiplex families.** Coordinates are given as GRCh37/hg19. HomozygosityMapper (HM) score is related to length of HBD block and number of shared alleles in the family^47^**.** We have also used a size threshold of ≥ 1 Mb for HBD regions, as has been used previously^36^.

| **Chr** | **start SNP** | **end SNP** | **start bp** | **end bp** | **size Mb** | **HM Score** |
| --- | --- | --- | --- | --- | --- | --- |
| **PKASD04** | | | | | | |
| 1 | rs1418440 | rs3010323 | 57290659 | 59567263 | 2.277 | 256 |
| 1 | rs9436444 | rs835342 | 50589798 | 53064034 | 2.474 | 94 |
| 3 | rs7629490 | rs1458006 | 87241497 | 88461825 | 1.22 | 104 |
| 4 | rs17686529 | rs6846723 | 166267383 | 167837707 | 1.57 | 181 |
| 6 | rs197988 | rs9379671 | 23593375 | 24614392 | 1.021 | 160 |
| 8 | rs7832023 | rs16893344 | 132370660 | 134206279 | 1.836 | 185 |
| 9 | rs11244336 | rs11137376 | 134118740 | 141025328 | 6.907 | 254 |
| 9 | rs10816710 | rs12003641 | 111511992 | 114279497 | 2.768 | 257 |
| 11 | rs11025963 | rs753688 | 21227542 | 44360545 | 23.133 | 257 |
| 13 | rs6491182 | rs2025237 | 27615791 | 37305641 | 9.69 | 256 |
| 14 | rs17119380 | rs4900375 | 84460068 | 98229487 | 13.769 | 256 |
| 18 | rs1116701 | rs11080474 | 9746909 | 11048468 | 1.302 | 257 |
| 20 | rs6139074 | rs1018493 | 63244 | 3,682,011 | 3.619 | 350 |
| **PKASD13** | | | | | | |
| 1 | rs11579207 | rs680285 | 92203190 | 93489113 | 1.286 | 7 |
| 1 | rs10493887 | rs4839069 | 97410506 | 111137811 | 13.727 | 8 |
| 2 | rs1354905 | rs785260 | 189446471 | 191020013 | 1.574 | 8 |
| 2 | rs6713102 | rs6745660 | 197174387 | 198349536 | 1.175 | 8 |
| 6 | rs1539348 | rs9343101 | 73710903 | 74739868 | 1.029 | 8 |
| 8 | rs12548521 | rs7822849 | 119552755 | 121827840 | 2.275 | 9 |
| 10 | rs914700 | rs1870519 | 45467703 | 47596804 | 2.129 | 65 |
| 11 | rs11224705 | rs361266 | 101176069 | 103937879 | 2.762 | 12 |
| 12 | rs254694 | rs2630218 | 115589524 | 128872849 | 13.283 | 17 |
| 12 | rs6486559 | rs7294615 | 131103914 | 133168145 | 2.064 | 10 |
| 13 | rs1331533 | rs9550238 | 104378857 | 114494245 | 10.115 | 23 |
| 21 | rs2223080 | rs2832390 | 29801854 | 30914526 | 1.113 | 17 |
| 22 | rs4607032 | rs1640350 | 17841068 | 20210363 | 2.369 | 500 |
| **PKASD14** | | | | | | |
| 2 | rs13036193 | rs6735385 | 5700470 | 9237190 | 3.537 | 259 |
| 3 | rs322707 | rs11916758 | 25319260 | 45131916 | 19.813 | 258 |
| 3 | rs9842685 | rs12489864 | 96167389 | 97322741 | 1.155 | 3 |
| 5 | rs4569852 | rs819571 | 71441589 | 72828213 | 1.387 | 42 |
| 6 | rs1358980 | rs6910674 | 43764551 | 71722821 | 27.958 | 12 |
| 6 | rs12524612 | rs34156731 | 132093249 | 157777283 | 25.684 | 15 |
| 6 | rs750618 | rs4392700 | 158982921 | 162724247 | 3.741 | 11 |
| 7 | rs1375238 | rs255142 | 17566398 | 30754949 | 13.189 | 14 |
| 12 | rs1480082 | rs2712623 | 98676087 | 101613488 | 2.937 | 18 |
| **PKASD17** | | | | | | |
| 6 | rs9348836 | rs9471831 | 11227987 | 42252385 | 31.024 | 500 |
| 8 | rs6987070 | rs7293734 | 41877317 | 47338235 | 5.461 | 176 |
| 11 | rs1323860 | rs2176611 | 30035236 | 33982853 | 3.948 | 500 |
| 17 | rs4646410 | rs9889937 | 17408699 | 18571366 | 1.163 | 80 |
| 17 | rs8072510 | rs8067765 | 33772658 | 34815551 | 1.043 | 140 |
| 17 | rs1230094 | rs4968280 | 43470156 | 44930363 | 1.460 | 130 |
| **PKASD25** | | | | | | |
| 7 | rs1718582 | rs2178044 | 68664061 | 70223193 | 1.559 | 5 |
| 8 | rs16938118 | rs1117213 | 49514893 | 51284360 | 1.769 | 82 |
| 10 | rs7086393 | rs1505908 | 58250470 | 59433399 | 1.183 | 11 |
| 11 | rs10838698 | rs9667626 | 47385923 | 48485877 | 1.1 | - |
| 16 | rs149156 | rs11075692 | 66912706 | 68681792 | 1.769 | 9 |
| **PKASD26** | | | | | | |
| 1 | rs2297684 | rs4653002 | 32146764 | 33770526 | 1.624 | 83 |
| 2 | rs11690543 | rs7597006 | 106037635 | 145209076 | 39.171 | 15 |
| 6 | rs3734694 | rs9349331 | 44118612 | 45808357 | 1.69 | 11 |
| 6 | rs2812152 | rs9353764 | 67143902 | 91529722 | 24.386 | 8 |
| 6 | rs10457161 | rs9400534 | 107505241 | 112661761 | 5.157 | 10 |
| 7 | rs12539724 | rs10808024 | 108358839 | 139763994 | 31.405 | 15 |
| 8 | rs10105278 | rs924184 | 26600068 | 28296171 | 1.696 | 257 |
| 8 | rs7815781 | rs10216833 | 109888569 | 123989445 | 14.101 | 254 |
| 8 | rs6577898 | rs2958522 | 139247027 | 146024952 | 6.778 | 257 |
| 10 | rs332114 | rs6481463 | 28925701 | 61102489 | 32.177 | 19 |
| 11 | rs4322381 | rs7931829 | 44741200 | 87248455 | 42.507 | 14 |
| 14 | rs11845309 | rs1980615 | 66513063 | 67958372 | 1.445 | 6 |
| 16 | rs12325368 | rs3112594 | 25776919 | 52671529 | 26.895 | 256 |
| 21 | rs7276198 | rs2827819 | 19582240 | 24440785 | 4.859 | 257 |
| **PKASD34 (N.B. affected sibs are monozygotic twins)** | | | | | | |
| 1 | rs3121532 | rs7641084 | 49369206 | 51989591 | 2.620 | 202 |
| 1 | rs6699043 | rs4641034 | 154913021 | 156342917 | 1.430 | 180 |
| 2 | rs13385191 | rs992996 | 20888265 | 22036018 | 1.148 | 212 |
| 2 | rs7589293 | rs6717323 | 81583557 | 82769739 | 1.186 | 172 |
| 3 | rs1351043 | rs9840698 | 82739213 | 84037979 | 1.299 | 166 |
| 4 | rs1238741 | rs3737483 | 99983312 | 101114999 | 1.430 | 278 |
| 5 | rs12517650 | rs7725903 | 20553425 | 21575563 | 1.022 | 122 |
| 5 | rs2619728 | rs1501908 | 155128570 | 156398169 | 1.270 | 184 |
| 6 | rs628629 | rs9450163 | 83894382 | 85818790 | 1.924 | 154 |
| 10 | rs17100087 | rs4402239 | 84213251 | 85276201 | 1.063 | 230 |
| 10 | rs2689700 | rs12769370 | 95946520 | 97032612 | 1.086 | 208 |
| 11 | rs6483110 | rs529911 | 91047120 | 92320830 | 1.274 | 162 |
| 14 | rs10138962 | rs17112078 | 104995893 | 107231967 | 2.048 | 162 |
| 15 | rs3825786 | rs7165146 | 42431142 | 44951174 | 2.520 | 250 |
| 16 | rs12445911 | rs4405548 | 47018079 | 49152331 | 2.134 | 122 |

**Table S2: Primer sequences for Sanger sequencing validation of variants (5’ to 3’)**

| **Primer name** | **Amplicon size (bp)** | **Primer sequence (5' to 3')** |
| --- | --- | --- |
| CDKL5_F | 220 | ACATCTCTCTTCGGCCTCAA |
| CDKL5_R |  | CACAGAGGACACATGCCAAC |
| TTC19_F | 150 | AAATGGCTGATTATTGATTTTGC |
| TTC19_R |  | AATGCCCAGATAATGGGTTTC |
| ZNF292_F | 211 | TTTGTACAGTTTGCCAATCCA |
| ZNF292_R |  | CTCTGCCTGTGGCTTTACCTA |
| SLC35A2_F | 223 | CCTCACTTCACCAGCACTGA |
| SLC35A2_R |  | ACCCATTATTTGCCCTTGG |
| NEDD4_F | 200 | CATTTACCAAGACCAAAGGAAA |
| NEDD4_R |  | TGGGAGGAGCTTATCCAAAT |
| EFCAB12_F | 197 | GGCACAATATCTGTGGCTGA |
| EFCAB12_R |  | TCGACACACATTCACACACG |
| PDZD4_F | 249 | GGCCAGCAGAGAGTCCAT |
| PDZD4_R |  | TAGCCCGGAAACGAAGAG |
| ARAP1_F | 342 | CAGTAGGCTCTTGGTGGGACA |
| ARAP1_R |  | GAGGATGGCGGTTGATGGTG |
| CSMD2_F | 223 | CCCTGAGAGAAAATTGGTTGG |
| CSMD2_R |  | CTTAACATGAGGCCCCTGAA |
| TMEM214_F | 229 | TATTAAGGGCTGGCAGATGG |
| TMEM214_R |  | CTGGGTGTCAGGGAGTTGTT |
| POLR3G_F | 249 | TTTCAGAATTTGCCCACTCA |
| POLR3G_R |  | GCTATAATCATACATCCAGTCCCATA |
| TMEM232_F | 219 | TGTTTTGATCCTAAAACAGATGTCA |
| TMEM232_R |  | TCTCTGGACCATGCTTCCTT |
| TRANK1a_F | 270 | GCAAGAACCTCTGAACTACTTCC |
| TRANK1a_R |  | CAGCAAGTCACACTGCACAG |
| TRANK1b_F | 267 | TGCCACTACTCACGCTTAAC |
| TRANK1b_R |  | TCCACACTGACTTGCTTTGC |
| EIF3H_F | 207 | AGCACTCACAAGGCCATCTA |
| EIF3H_R |  | CACGCGAGACTTGTACTTCC |
| GML_F | 286 | ATTCAGGTGGGCAGAGTCAG |
| GML_R |  | CAGCCAATCATCCCAGTGAC |

**Figure S1: Pedigrees and electropherograms for all reported variants.** Blue bar in electropherogram corresponds to the position of the sequence variant. In some cases where indicated, supporting evidence from whole exome sequence (WES) using the Integrated Genome Browser (IGV) was available where Sanger sequence was not.

**
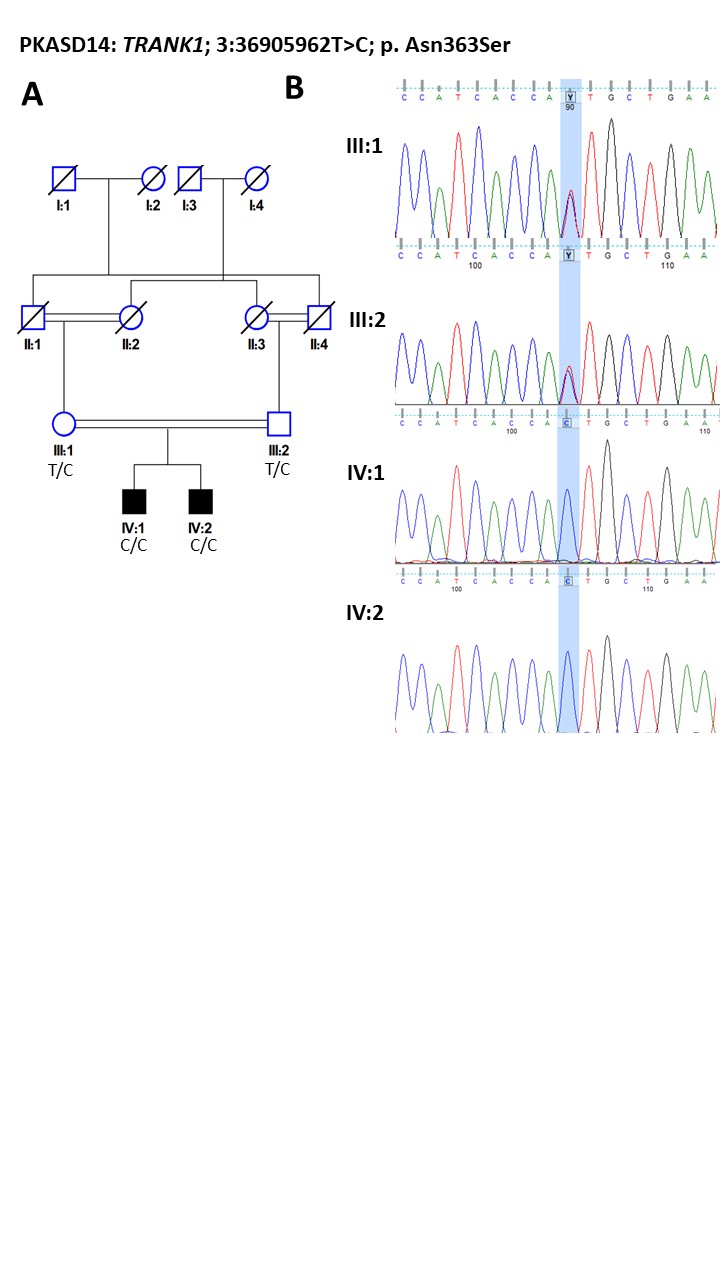
**

**
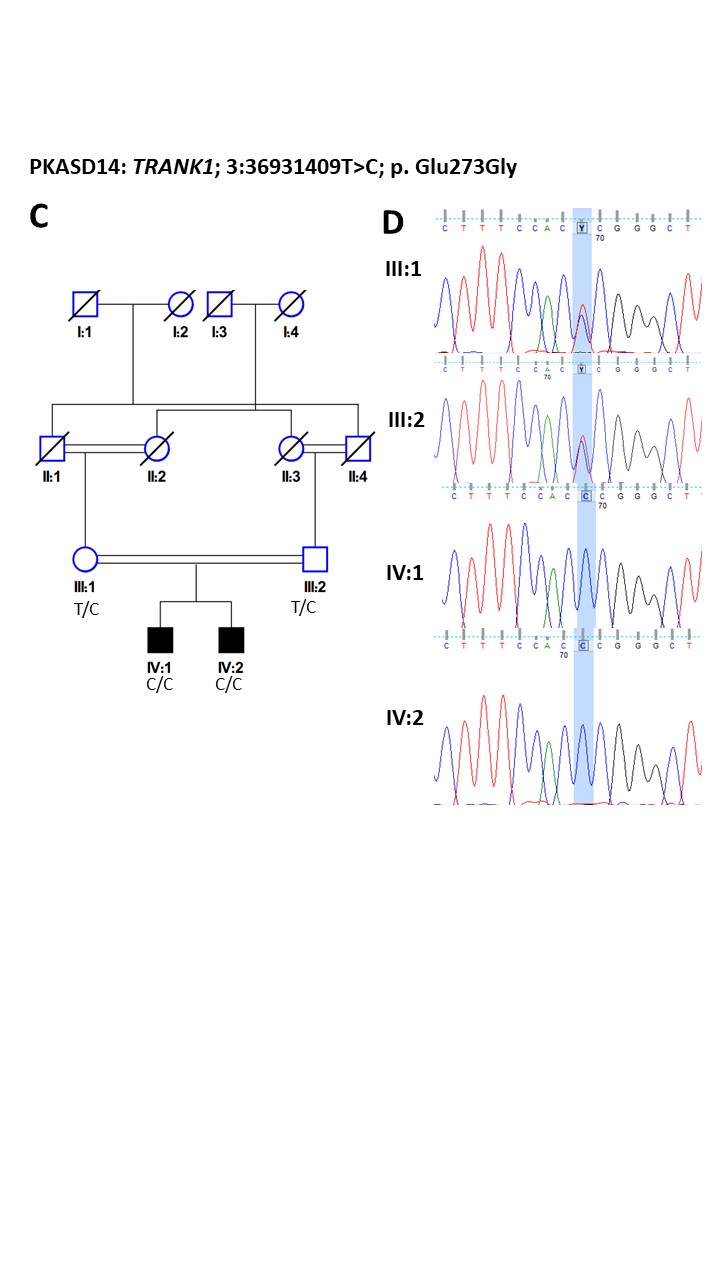
**

**
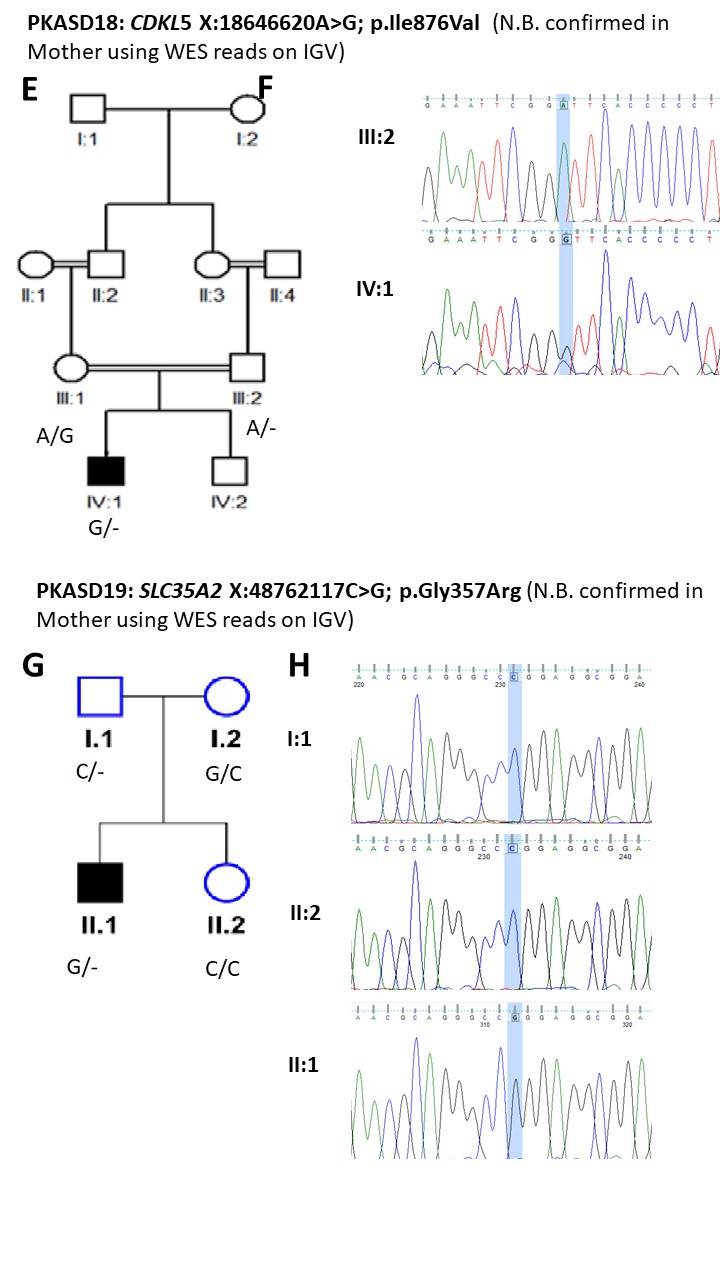
**

**
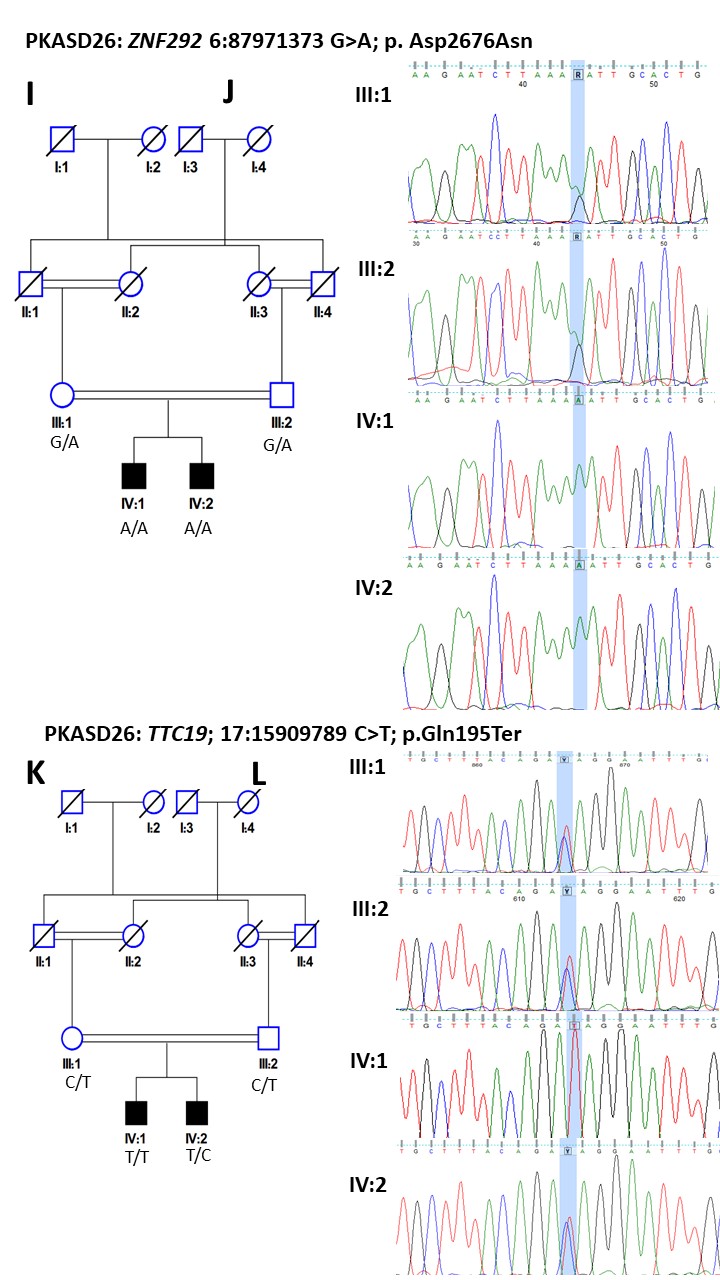
**

**
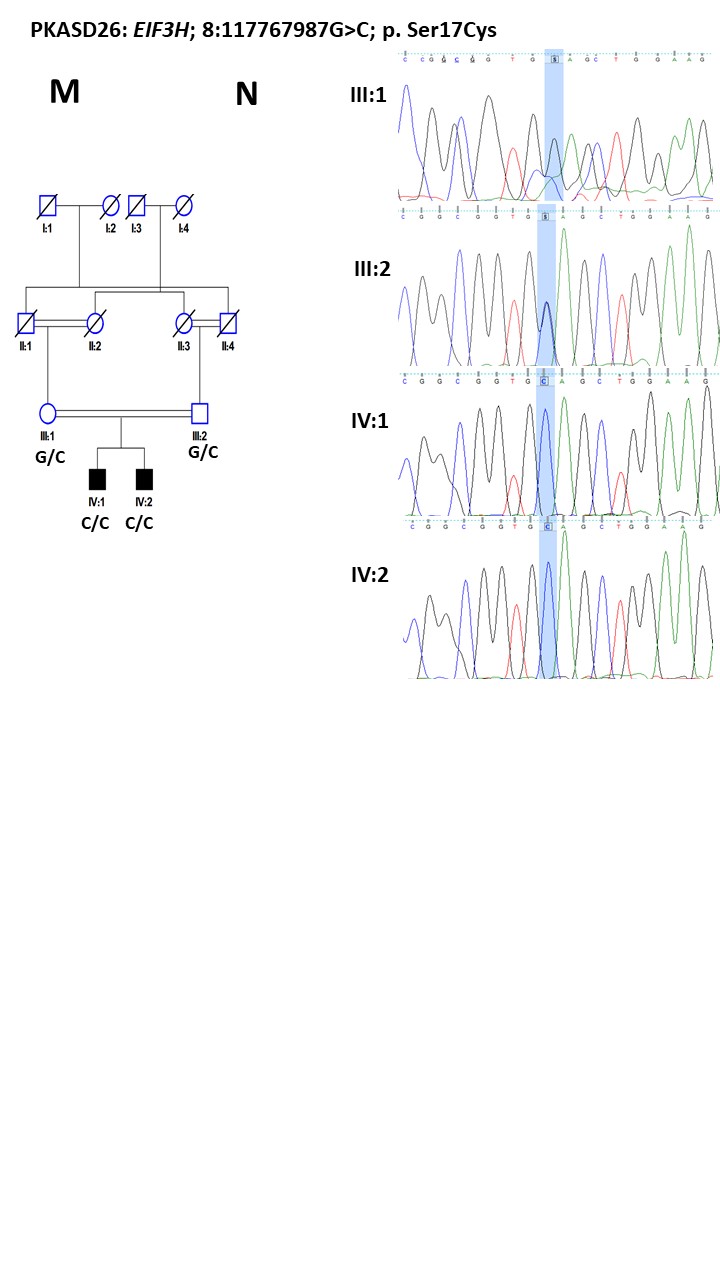
**

**
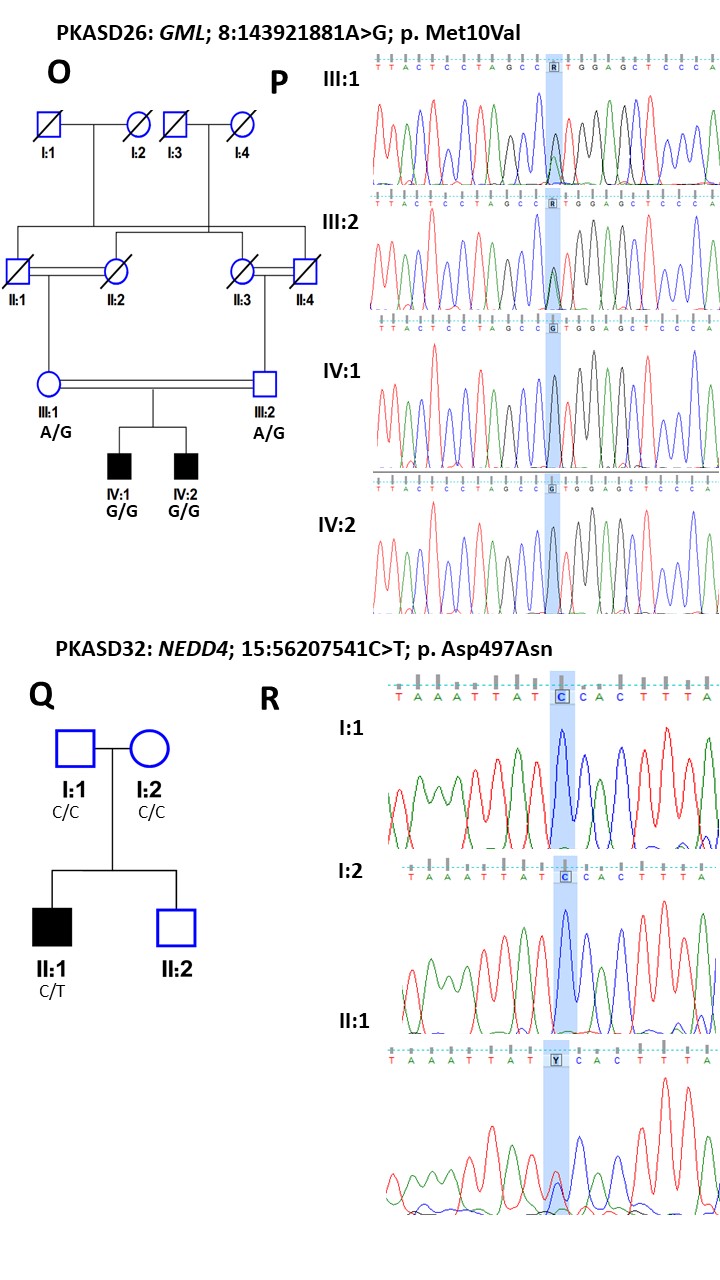
**

**
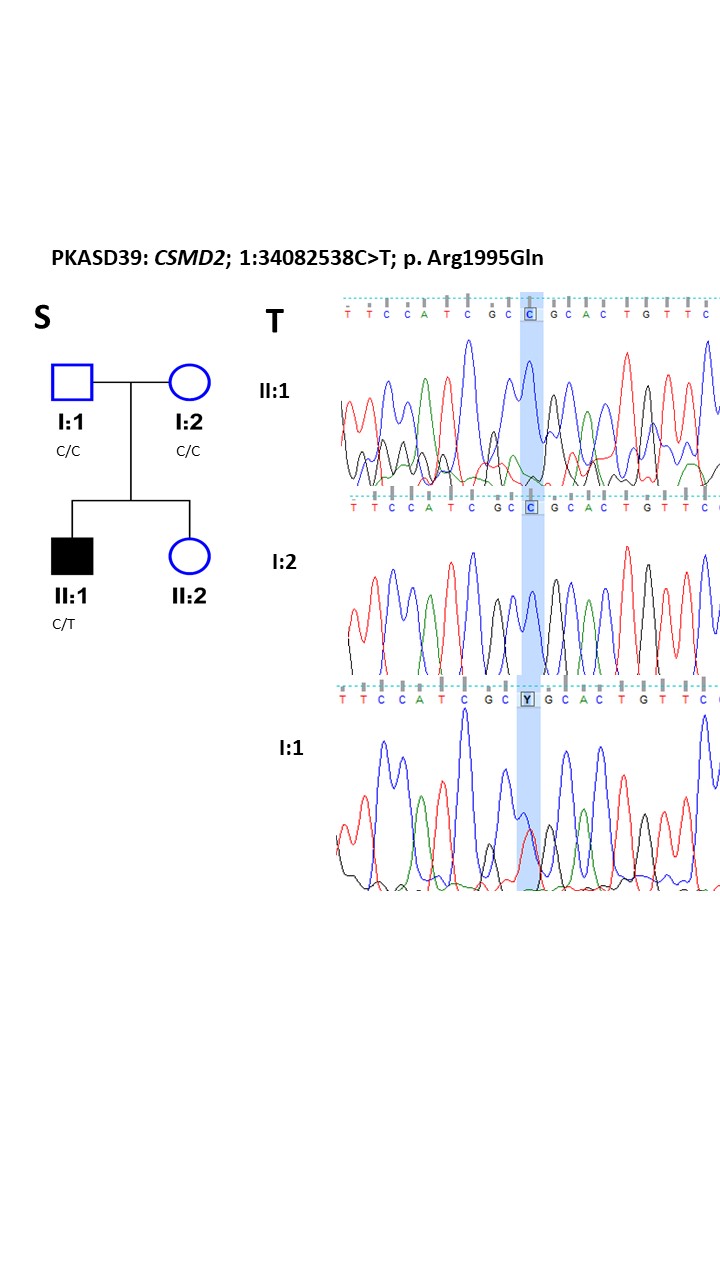
**

**
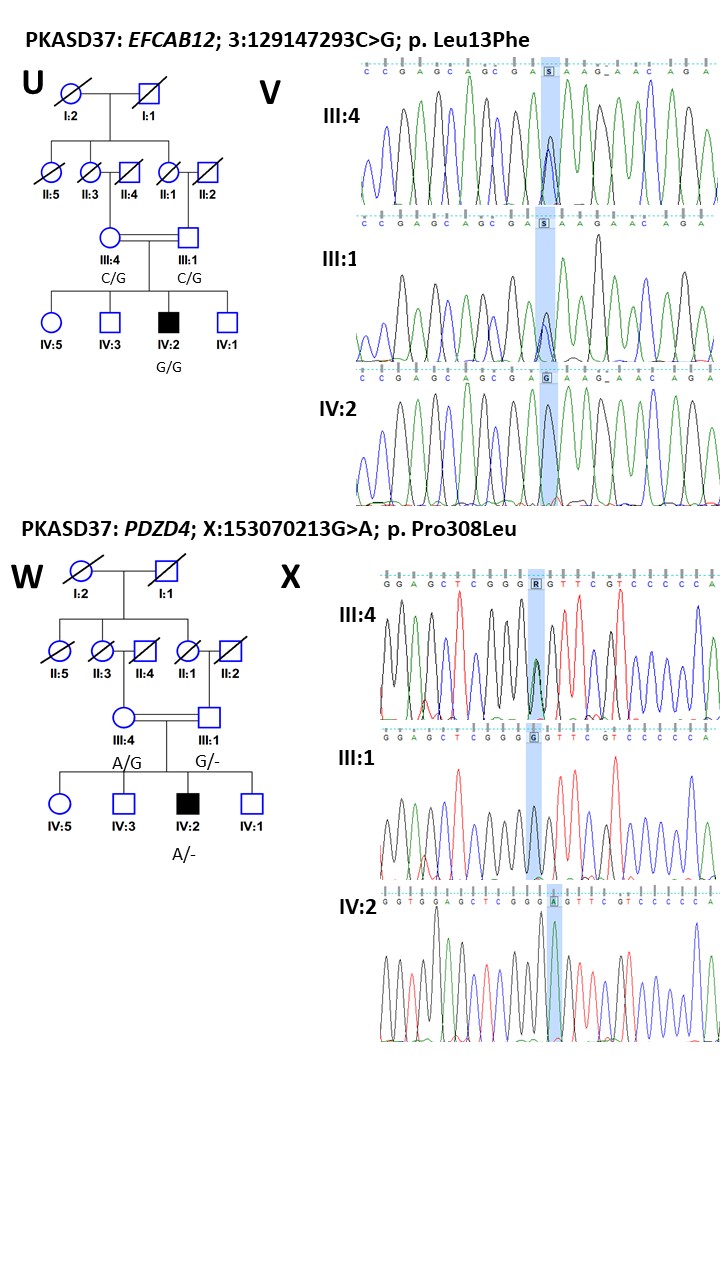
**

**
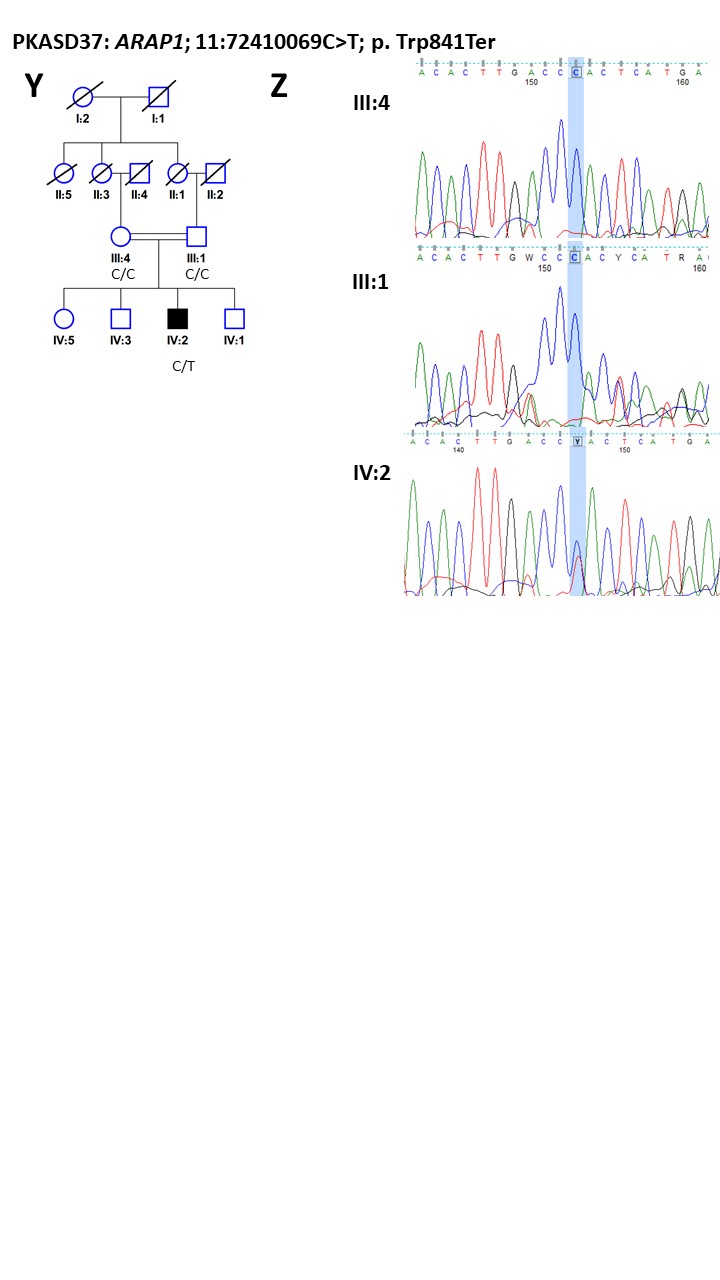
**

**
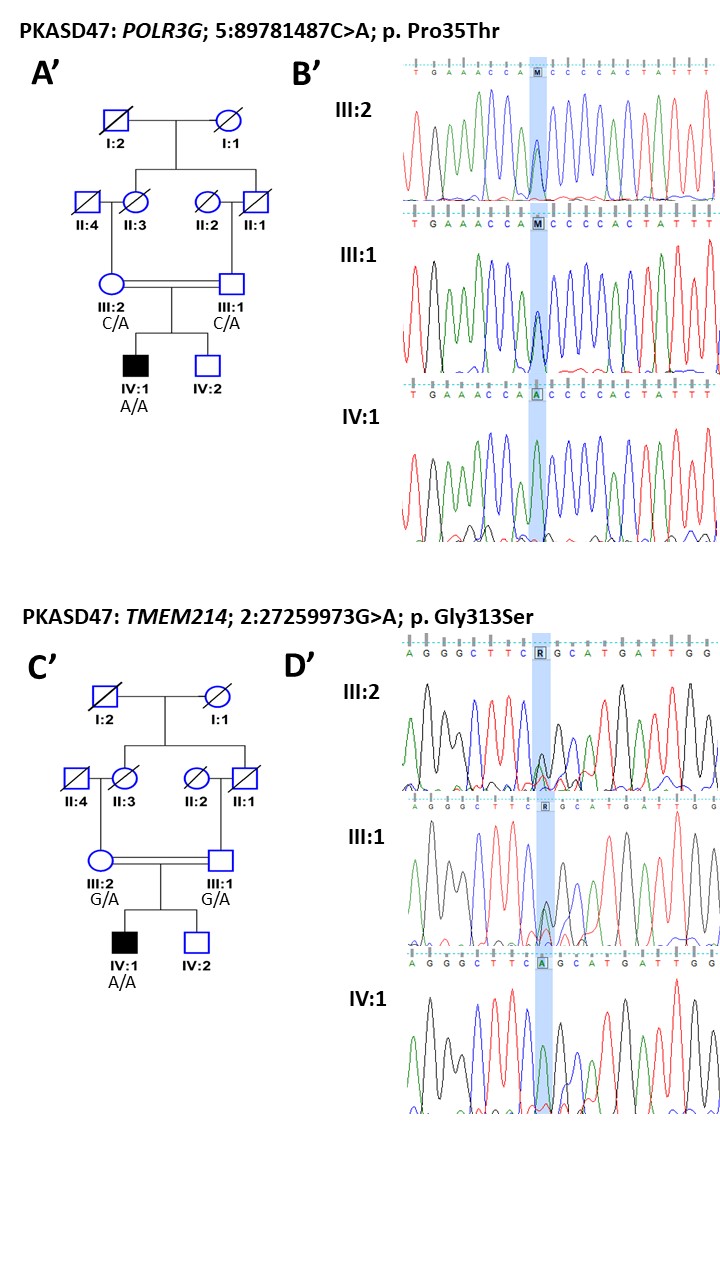
**

**
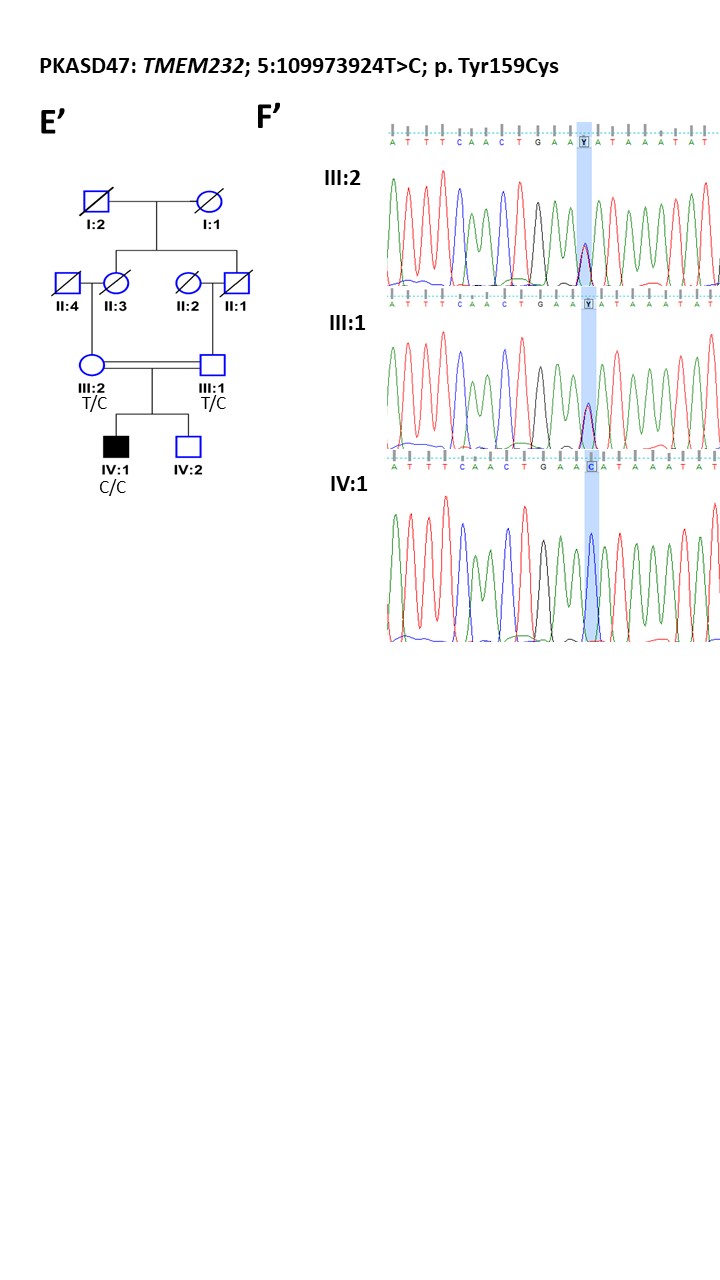
**

**
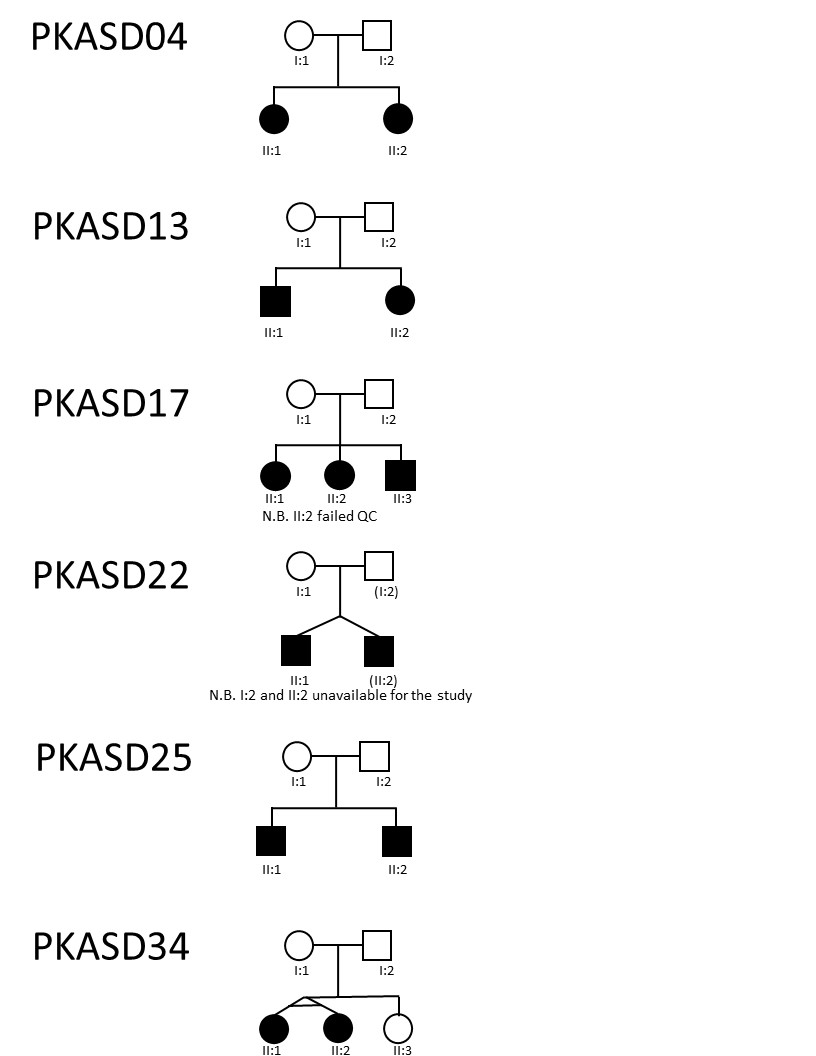
**

**Table S3: Candidate variants:** Genomic coordinates are given relative to GRCh37/hg19 build. gnomAD minor allele frequency (MAF) v2.1.1 is provided, with number of homozygotes or hemizygotes for X-linked genes given in parentheses. * indicates the variant is located within an HBD block (in a multiplex family). Candidate genes were cross-referenced with the MSSNG dataset ((N>11,500 individuals; research.mss.ng; accessed Aug 2023), as well as the Autism Sequencing Consortium dataset (asc.broadinstitute.org; accessed Aug 2023). For MSSNG, high quality coding change variants, either *de novo*, homozygous (autosomal), or hemizygous (X-chromosomal) that were rare or absent in the gnomAD control datasets (accessed Aug 2023), and with no hemizygotes or homozygotes among the gnomAD controls, are indicated.

| Family ID | Sex | Chromosome | Gene | Protein variant | Mutation type | Mutation effect | gnomAD MAF all | gnomAD MAF S. Asian | ASC case | ASC control | MSSNG case |
| --- | --- | --- | --- | --- | --- | --- | --- | --- | --- | --- | --- |
| PKASD-14 | M | 3:36905962T>C* | TRANK1 | Asn363Ser | Homo | missense | 2.99E-05 (0) | 2.00E-04 (0) | 0 | 0 | No homozygous or *de novo* |
|  |  | 3:36931409T>C* | TRANK1 | Glu273Gly | Homo | missense | 7.62E-04 (0) | 4.84E-04 (0) | 0 | 0 | No homozygous or *de novo* |
| PKASD-18 | M | X:18646620A>G | CDKL5 | Ile876Val | XL | missense | 0 | 0 | 1 (splice acceptor) | 0 | 3 missense hemi(M):  NM_003159:c.16A>G:p.I6V (no hemis in gnomAD)  c.92G>A:p.R31K (no hemis in gnomAD); c.2572C>T:p.R858C (no hemis in gnomAD);  1 frameshift del het(F):  NM_003159:c.2635_2636del:p.L879fs (no hemis in gnomAD) |
| PKASD-19 | M | X:48762117C>G | SLC35A2 | Gly357Arg | XL | missense | 1.1E-05 (1) | 5.304E-05 (1) | 0 | 0 | 2 affected brothers with missense hemizygous ( maternal): NM_005660:c.109T>C:p.Y37H (no alleles in gnomAD controls) |
| PKASD-26 | M | 17:15909789C>T | TTC19 | Gln195* | Homo | Stop gain | 3.18E-05 (0) | 3.266E-05 (0) | 0 | 0 | No homozygous or *de novo* |
|  |  | 6:87971373G>A* | ZNF292 | Asp2676Asn | Homo | missense | 3.891E-04 (0) | 3.486E-03 (0) | 1 | 0 | 1 missense homo: NM_015021:c.6946C>T:p.R2316W  0 *de novo* |
|  |  | 8:117767987G>C* | EIF3H | Ser17Cys | Homo | missense | 7.562E-05 (0) | 6.206E-04 (0) | 0 | 1 | No homozygous or *de novo* |
|  |  | 8:143921881A>G* | GML | Met10Val | Homo | missense | 4.95E-05 (0) | 1.96E-04 (0) | 0 | 0 | No homozygous or *de novo* |
| PKASD-32 | M | 15:56207541C>T | NEDD4 | Asp497Asn | De novo | missense | 0 | 0 | 0 | 0 | No homozygous or *de novo* |
| PKASD-37 | M | 3:129147293C>G | EFCAB12 | Leu13Phe | Homo | missense | 1.871E-4 (0) | 1.489E-3 (0) | 0 | 0 | No homozygous or *de novoh* |
|  |  | X:153070213G>A | PDZD4 | Pro308Leu | XL | missense | 5.5E-06 (0) | 0 | 0 | 0 | 2 missense hemi (M):  NM_032512:c.1334C>T:p.A445V (no hemis in gnomAD) NM_032512:c.976G>A:p.D326N (no hemis in gnomAD) |
|  |  | 11:72410069C>T | ARAP1 | Trp841* | De novo | Stop gain | 0 | 0 | 0 | 0 | No homozygous or *de novo* |
| PKASD-39 | M | 1:34082538C>T | CSMD2 | Arg1995Gln | De novo | missense | 0 | 0 | 1 *de novo* (c.24delC; p.G9Afs*303) | 0 | No homozygous (except where homozygotes also in gnomAD controls) or *de novo* |
| PKASD-47 | M | 2:27259973G>A | TMEM214 | Gly313Ser | Homo | missense | 1.21E-04 (0) | 8.496E-04 (0) | 3 *de novo* (c.499C>A; p.H167N; c.661A>G; p.I221V; c.1654T>C; p.S552P) | 0 | No homozygous or *de novo* |
|  |  | 5:89781487C>A | POLR3G | Pro35Thr | Homo | missense | 1.429E-04 (0) | 2.973E-04 (0) | 0 | 0 | No homozygous or *de novo* |
|  |  | 5:109973924T>C | TMEM232 | Tyr159Cys | Homo | missense | 0 | 0 | 0 | 0 | No homozygous or *de novo* |

**Table S4: Consanguinity through microarray genotype analysis.** Runs of homozygosity (RoH) was also calculated from autosomal genotype data, using PLINK 1.9, and used to generate an F-coefficient of consanguinity for the probands (and affected siblings), F_RoH_, as described in McQuillan et al, 2008,^48^ where F_roh_ = ∑ L_roh_/L_auto_. Autosome size L_auto_ estimated at 2,673,768 Kb**.** Relatedness of parents: Identity-by-descent estimation using Pi_Hat scores provided by PLINK analysis of microarray genotype data, where available. UA= unaffected sibling.

| Child ID | Σ_LRoH_ (Kb) | F_RoH_ | Mother ID | Father ID | PI_HAT | Approximate Relatedness of parents |
| --- | --- | --- | --- | --- | --- | --- |
| PKASD-01_C | 45691 | 0.017 |  |  |  |  |
| PKASD-02_C | 203338 | 0.076 | PKASD-02_M | PKASD-02_F | 0.1144 | 3^rd^ degree |
| PKASD-03_C | 31162 | 0.012 | PKASD-03_F | PKASD-03_M | 0 | parents unrelated |
| PKASD-04_C1 | 196924 | 0.074 | PKASD-04_M | PKASD-04_F | 0.2279 | 2^nd^ degree |
| PKASD-04_C2 | 331829 | 0.124 |  |  |  |  |
| PKASD-05_C | 50148 | 0.019 | PKASD-05_M | PKASD-05_F | 0.0033 | distantly related |
| PKASD-06_C | 278727 | 0.104 | PKASD-06_M | PKASD-06_F | 0.0765 |  |
| PKASD-07_C | 46179 | 0.017 | PKASD-07_M | PKASD-07_F | 0 | parents unrelated |
| PKASD-08_C | 46509 | 0.017 | PKASD-08_M | PKASD-08_F | 0 | parents unrelated |
| PKASD-10_C | 42287 | 0.016 | PKASD-10_M | PKASD-10_F | 0 | parents unrelated |
| PKASD-11_C | 57522 | 0.022 | PKASD-11_M | PKASD-11_F | 0 | parents unrelated |
| PKASD-13_C1 | 226114 | 0.085 |  |  |  |  |
| PKASD-13_C2 | 142320 | 0.053 |  |  |  |  |
| PKASD-14_C1 | 229569 | 0.086 | PKASD-14_M | PKASD-14_F | 0.1266 | 3^rd^ degree |
| PKASD-14_C2 | 339961 | 0.127 |  |  |  |  |
| PKASD-15_C | 35629 | 0.013 | PKASD-15_M | PKASD-15_F | 0 | parents unrelated |
| PKASD-16_C | 152861 | 0.057 | PKASD-16_M | PKASD-16_F | 0.1353 | 2^nd^ to 3^rd^ degree |
| PKASD-17_C | 256631 | 0.096 | PKASD-17_M | PKASD-17_F | 0.1719 | 2^nd^ to 3^rd^ degree |
| PKASD-18_C | 43655 | 0.016 | PKASD-18_M | PKASD-18_F | 0.0159 | distantly related |
| PKASD-19_C1 | 188988 | 0.071 | PKASD-19_M | PKASD-19_F | 0 | parents unrelated |
| PKASD-19_C2 (UA) | 36879 | 0.014 |  |  |  |  |
| PKASD-20_C | 56993 | 0.021 | PKASD-20_M | PKASD-20_F | 0.0121 | distantly related |
| PKASD-21_C | 38192 | 0.014 |  |  |  |  |
| PKASD-22_C2 | 244335 | 0.091 |  |  |  |  |
| PKASD-25_C1 | 41811 | 0.016 | PKASD-25_M | PKASD-25_F | 0 | parents unrelated |
| PKASD-25_C2 | 43132 | 0.016 |  |  |  |  |
| PKASD-26_C1 | 498088 | 0.186 | PKASD-26_F | PKASD-26_M | 0.2278 | 2^nd^ degree (1^st^ cousin marriage) |
| PKASD-26_C2 | 458022 | 0.171 |  |  |  |  |
| PKASD-27C | 42965 | 0.016 | PKASD-27_F | PKASD-27_M | 0 | parents unrelated |
| PKASD-28C | 55873 | 0.021 | PKASD-28_M | PKASD-28_F | 0 | parents unrelated |
| PKASD-29C | 52767 | 0.020 | PKASD-29_M | PKASD-29_F | 0 | parents unrelated |
| PKASD-30C | 42627 | 0.016 | PKASD-30_M | PKASD-30_F | 0 | parents unrelated |
| PKASD-31C | 501066 | 0.187 |  |  |  |  |
| PKASD-32C | 44753 | 0.017 | PKASD-32_M | PKASD-32_F | 0 | parents unrelated |
| PKASD-33_C | 254375 | 0.095 | PKASD-33_M | PKASD-33_F | 0.1797 | 2^nd^ to 3^rd^ degree |
| PKASD-34_C1 | 40999 | 0.015 | PKASD-34_M | PKASD-34_F | 0 | parents unrelated |
| PKASD-34_C2 | 42053 | 0.016 |  |  |  |  |
| PKASD-34_C3 (UA) | 44267 | 0.017 |  |  |  |  |
| PKASD-35_C | 255486 | 0.096 | PKASD-35_M | PKASD-35_F | 0.1648 | 2^nd^ to 3^rd^ degree |
| PKASD-36_C1 | 39648 | 0.015 | PKASD-36_M | PKASD-36_F | 0 | parents unrelated |
| PKASD-36_C2 (UA) | 50993 | 0.019 |  |  |  |  |
| PKASD-37_C | 70853 | 0.026 | PKASD-37_M | PKASD-37_F | 0 | parents unrelated |
| PKASD-38_C1 | 35221 | 0.013 | PKASD-38_F | PKASD-38_M | 0 | parents unrelated |
| PKASD-38_C2 (UA) | 55717 | 0.021 |  |  |  |  |
| PKASD-39C | 39157 | 0.015 | PKASD-39_F | PKASD-39_M | 0 | parents unrelated |
| PKASD-47_C | 431250 | 0.161 | PKASD-47_F | PKASD-47_M | 0.1588 | 2^nd^ to 3^rd^ degree |
